# Animal Venoms and Their Components: Molecular Mechanisms of Action

**DOI:** 10.3390/toxins13060415

**Published:** 2021-06-11

**Authors:** Yuri Utkin

**Affiliations:** Laboratory of Molecular Toxinology, Shemyakin-Ovchinnikov Institute of Bioorganic Chemistry, Russian Academy of Sciences, 117997 Moscow, Russia; utkin@ibch.ru or yutkin@yandex.ru; Tel.: +7-495-3366522

Animal venoms comprise numerous toxin families, consisting mainly of peptides and proteins. In prey, these toxins affect various vitally important systems that may result in severe illness or death. During evolution, toxins acquired the ability to bind selectively and with high affinity to different biological targets in organisms. However, at present, not all toxin targets have been identified, and not all the molecular mechanisms underlying the effects of toxins are understood. This understanding is very important for the efficient treatment of envenomation, which still continues to be a significant problem. On the other hand, the high selectivity and efficiency of toxins make them valuable molecular tools for fundamental research. Moreover, toxins with known mechanisms of action may serve as templates for drug development. All this suggests that studying the molecular mechanisms of action of animal venoms and their toxins is a very challenging and important task. The aim of this Special Issue of *Toxins* is to present a modern understanding of the various aspects of the molecular mechanisms underlying the action of animal venoms and their constituents.

Structures and molecular mechanisms of action vary greatly among toxins from different animals. Venomous animals can be found among representatives of various types, classes, orders, families and genera. This diversity is reflected in the articles published in this Special Issue. There are articles that deal with the toxins from insects, spiders, scorpions, octopuses, toads, frogs, and snakes. The topics range from initial characterization of biological activity of toxins to detailed investigation of the mechanism of action at the level of viruses, cells, organs, or live animal. Different methods to study the biological activity were applied, including molecular modeling, electrophysiology, spectroscopy (NMR), molecular biology etc.

In this Special Issue, several articles are focused on the characterization of venoms and poisons from species not studied earlier. Thus, three papers describe the effects of toad poisons. For example, in the parotoid gland secretion of the toad *Rhinella horribilis*, more than 50 compounds (including alkaloids, bufadienolides, and argininyl diacids derivatives) are identified using HPLC-MS/MS [1]. The secretion and one of its fractions manifest antiproliferative activity against A549 cancer cells. The active fraction strongly inhibits cancer cell migration as well. The investigation of the parotoid secretion from another toad species *R. schneideri* demonstrates an insecticidal activity towards *Nauphoeta cinerea* cockroaches [2]. Together with an earlier finding of entomotoxicity for the *R. icterica* secretion done by the same group [3], this may lead to the development of new ecologically safe insecticides. The secretion of *R. icterica* is also studied using avian preparations [4]. After separation of the secretion via reversed phase HPLC, several fractions are obtained, one of which reproduced the biological effects of the crude secretion and contained bufadienolides and cardiac glycosides. In the avian peripheral nervous system, this fraction shows interactions with several molecular targets, including acetylcholinesterase, the Na+/K+-ATPase pump and L-type Ca2+ channels, as well as stabilization of the cell membrane. This is the first indication of the effects of toad poison on avian tissues.

One paper focuses on the molecular evolution of three-finger toxins (3FTX) in the snake genus *Calliophis*, which is the most basal branch of the family Elapidae [5]. It is shown that this genus produces the classic elapid eight-cysteine 3FTXs, which form several different clades. The venom of one of the *Calliophis* species (*C. bivirgatus*) contains a unique toxin (calliotoxin) that acts on the voltage-gated sodium (Na_V_) channels. Based on the analysis done in the study, it was hypothesized that elongated glands in *Calliophis* species may have evolved together with Na_V_ channel toxins.

About one third of the Special Issue papers deal mainly with the structural and functional characterization of new toxins. These toxins were isolated from the venoms of snakes, scorpions, spiders, centipedes, and wasps, as well as the poison of a frog. Thus, the investigation of novel three-finger neurotoxins from *Naja melanoleuca* cobra venom revealed the structural elements involved in the interaction of toxins with nicotinic acetylcholine receptors (nAChRs) and GABA_A_ receptors [6]. The results obtained suggest that toxin loop III may participate in the effective interaction of some long-chain neurotoxins with a GABA_A_ receptor. From the venom of the scorpion *Buthus martensii,* the new toxin BmK NSPK was isolated [7]. The toxin inhibits outward K+ current without affecting sodium channel, depolarized membrane, and increased spontaneous calcium oscillation in spinal cord neurons. Moreover, it manifests quite interesting biological activity, promoting neurite outgrowth through the NGF/TrkA signaling pathway. Two new toxins inhibiting tetrodotoxin-sensitive sodium channels are isolated from the venom of the spider *Cyriopagopus longipes* and characterized [8]. Among several sodium channels investigated, the toxins showed the highest activity against NaV1.7 subtype. These toxins may be used as molecular tools for the pharmacological study of sodium channels.

In a comparative study, the venom of the Chilean *Sicarius thomisoides* spider is characterized with that of the *Loxosceles laeta* spider [9]. The electrophoretic patter, as well as hemolytic and cytotoxic activities, is compared and shows some similarity. Phospholipase D enzymatic activity was characteristic of both venoms. Basing on the data obtained, it was concluded that *S. thomisoides* venom can seriously harm people. An acyl-activating enzyme (4-coumarate:CoA ligase-like protein) was cloned from the venom apparatus of the endoparasitoid wasp *Tetrastichus brontispae* (Hymenoptera: *Eulophidae*) [10]. A recombinant enzyme manifested insecticidal activity against a hispid *Octodonta nipae* that damages several species of palm trees. The molecular mechanism of the enzyme activity may involve the regulation of host immunity through fatty acid metabolism-derived signaling pathways. From the cDNA library of the venom glands of centipede *Scolopendra hainanum*, a kazal-type serine protease inhibitor (ShSPI) was identified [11]. ShSPI efficiently inhibited porcine pancreatic elastase and human neutrophils elastase, with *Ki* values of 225.8 nM and 12.6 nM, respectively. As elastases are involved in several important physiological cascades, the identified inhibitor may be regarded as a candidate to develop a drug for cardiopulmonary diseases. From the poison of the frog *Amolops jingdongensis*, a prokineticin homolog Bv8-A was isolated and characterized [12]. Bv8-AJ accelerated wound healing in mice, presumably by exerting potent proliferative effects on fibroblasts and keratinocytes.

A great deal of papers in the Special Issue are focused on the molecular mechanisms underlying biological effects of animal venoms and toxins. The investigated effects are very diverse and ranged from antiviral and anticancer activities to hyperglycemia caused by scorpion envenomation. Thus, a life-threatening transitory hyperglycemia is among the *Tityus serrulatus* scorpion’s envenomation effects. The study aimed to investigate that hyperglycemia due to scorpion envenomation may involve inflammatory signaling in the pancreas showed that *T. serrulatus* venom indeed induced the production of IL-1α and IL-1β in the pancreas, which provoked nitric oxide (NO) production and edema in β-cells in islets [13]. The authors suggest that a supportive therapy with inhibitors of NO production may help to prevent fatal outcomes of scorpion envenomation. The analysis of the biological effects produced by the crude venom from the larval stage of *Latoia consocia* living in Southwest China showed that it induced severe acute pain behaviors after intraplantar injection into the hind paws of mice [14]. The studies on TRPV1-deficit (TRPV1 KO) mice and the use of the TRPV1-specific inhibitor capsazepine suggest that TRPV1 serves as a primary nociceptor in caterpillar-induced pain. To understand the molecular mechanisms involved in the prey immobilization resulting from *Protobothrops mucrosquamatus* and *Trimeresurus stejnegeri* venoms, C-type lectin-like proteins mucetin and stejnulxin isolated from these venoms were studied [15]. It was shown that both proteins induced acute cerebral ischemia and reduced animal locomotor activity. The presence of these toxins in *Protobothrops mucrosquamatus* and *Trimeresurus stejnegeri* venoms make them similar in immobilization activity to *Naja atra* venom, albeit through a different mechanism. The paper by Kuo et al. [16] describes the antithrombotic effect of peptide derived from snake venom disintegrin. To improve antithrombotic activity and diminish bleeding side effects, a RGD-containing fragment of disintegrin trimucrin was mutated from 50ARGDNP55 to 50AKGDRR55 and the latter was modified using N-terminal PEGylation technique. As a result, the derivative with a prolonged half-life and decreased bleeding risk was obtained and represents a safer antithrombotic agent for long-acting treatment of thrombus diseases.

Several papers describe effects of animal toxins or related peptides on microorganisms. Thus, the screening of snake venoms against protozoan *Tetrahymena pyriformis* revealed the high activity of cobra venoms [17]. Further analysis of the venoms showed that the active components are cytotoxins of the 3FTX family. The possible molecular mechanism of cytotoxin effects may include membrane rupture causing the death of ciliate *T. pyriformis.* A life-threatening infectious disease caused by parasitic protozoa of the genus *Plasmodium* (mainly *Plasmodium falciparum*) is malaria. To overcome antimalarial drug resistance (which has emerged as a global problem recently [18]), the development of novel drugs is in urgent need. In the work of Fang et al. [19], an antimicrobial peptide LZ1 derived from snake cathelicidin and possessing antimalarial activity was identified. The authors considered LZ1 as a potential candidate for novel antimalarial development. Other microorganisms transmitted by mosquitoes are flaviviruses, including such dangerous microorganisms as dengue virus (DENV), Japanese encephalitis (JEV), West Nile (WNV), and Zika virus (ZIKV). In the venom of the *Alopecosa nagpag* spider, a defense peptide Av-LCTX-An1a was identified, and its anti-dengue serotype-2 virus activity was studied [20]. It was found that An1a functioned as an inhibitor NS2B–NS3 proteases in DENV as well as ZIKV and might be a candidate for the design of anti-flaviviral drug.

The Special Issue did not remain without a description of such an important property of animal toxins as anticancer activity. To identify new drug candidates against melanoma, the molecular mechanism of action of the *Octopus kaurna*-derived peptide, Octpep-1, was investigated [21]. It was shown that Octpep-1 acted synergistically with rapamycin or an ERK inhibitor. These combinations lead to the enhancement of its antiproliferative profile and did not interfere with the proliferation of the healthy NFF cells. It was concluded that Octpep-1 might have the therapeutic potential for the treatment of melanoma.

Two papers in the Special Issue deal with the molecular mechanisms of interaction of 3FTXs with their targets. Thus, 3FTXs interaction with nAChR was studied via a homology modeling and protein docking protocol [22]. Utilizing the Rosetta protein–protein docking application, the authors adapted the previously [23] developed ToxDock protocol for 3FTX. The docking protocol was tested on eight toxins with known α7 and muscle-type nAChR binding properties and reasonably predicted the qualitative and some specific aspects of 3FTX binding to nAChR. In the other paper, the interaction of three-finger cardiotoxins from cobra venom with outer mitochondrial membrane was studied [24]. Using ^31^P-NMR and ^1^H-NMR spectroscopy, the effects of cardiotoxins CTI and CTII on phospholipid packing and dynamics in model phosphatidylcholine membranes that mimic a phospholipid composition in the outer mitochondrial membrane (OMM) was analyzed. To study the molecular mechanisms by which CTII and CTI bind to a phospholipid membrane, molecular dynamics and molecular docking were applied. As a result, a conceptual model that explains a molecular mechanism by which cobra venom cardiotoxins interact with mitochondrial membranes was proposed.

Quite an interesting molecular mechanism for the potentiation of venom toxicity to insect was discovered in the work by Clémençon et al. [25]. It was found that, according microscale thermophoresis data, insecticidal neurotoxins from the venom of the spider *Cupiennius salei* formed complexes. In addition, through the use of the voltage clamp technique in the oocytes of *Xenopus laevis*, it was shown that a combined application of two neurotoxins of different types has a much more pronounced cytolytic effect than each of the toxins alone, and toxicity to *Drosophila melanogaster* greatly increased at the combined application of two toxins. Thus, the high cytolytic activity of *Cupiennius salei* venom was explained by the merge of two different toxins.

In conclusion, the papers in the Special Issue addressing the different aspects of animal venoms contribute to a deeper understanding of their molecular composition and the biological activity of the constituent toxins. Some papers, including in particular those describing the antimicrobial and anticancer properties of toxins and their analogs, bring us closer to the development of new modern drugs. Other papers demonstrating the underlying molecular mechanisms of action of venoms and toxins reveal the fundamental basis for the functioning of various vital systems in living organisms. Thus, animal toxins have a large, not yet fully disclosed potential for use both as molecular tools for the study of physiological processes and as a basis for the design of new drugs. Both these spheres deserve further extensive and rigorous investigation.
